# A Machine Learning Approach to Assess Differential Item Functioning in Psychometric Questionnaires Using the Elastic Net Regularized Ordinal Logistic Regression in Small Sample Size Groups

**DOI:** 10.1155/2021/6854477

**Published:** 2021-12-15

**Authors:** Vahid Ebrahimi, Zahra Bagheri, Zahra Shayan, Peyman Jafari

**Affiliations:** Department of Biostatistics, School of Medicine, Shiraz University of Medical Sciences, Shiraz, Iran

## Abstract

Assessing differential item functioning (DIF) using the ordinal logistic regression (OLR) model highly depends on the asymptotic sampling distribution of the maximum likelihood (ML) estimators. The ML estimation method, which is often used to estimate the parameters of the OLR model for DIF detection, may be substantially biased with small samples. This study is aimed at proposing a new application of the elastic net regularized OLR model, as a special type of machine learning method, for assessing DIF between two groups with small samples. Accordingly, a simulation study was conducted to compare the powers and type I error rates of the regularized and nonregularized OLR models in detecting DIF under various conditions including moderate and severe magnitudes of DIF (DIF = 0.4 and 0.8), sample size (*N*), sample size ratio (*R*), scale length (*I*), and weighting parameter (*w*). The simulation results revealed that for *I* = 5 and regardless of *R*, the elastic net regularized OLR model with *w* = 0.1, as compared with the nonregularized OLR model, increased the power of detecting moderate uniform DIF (DIF = 0.4) approximately 35% and 21% for *N* = 100 and 150, respectively. Moreover, for *I* = 10 and severe uniform DIF (DIF = 0.8), the average power of the elastic net regularized OLR model with 0.03 ≤ *w* ≤ 0.06, as compared with the nonregularized OLR model, increased approximately 29.3% and 11.2% for *N* = 100 and 150, respectively. In these cases, the type I error rates of the regularized and nonregularized OLR models were below or close to the nominal level of 0.05. In general, this simulation study showed that the elastic net regularized OLR model outperformed the nonregularized OLR model especially in extremely small sample size groups. Furthermore, the present research provided a guideline and some recommendations for researchers who conduct DIF studies with small sample sizes.

## 1. Introduction

In psychometric research such as health-related quality of life (HRQoL), measurement invariance, also known as differential item functioning (DIF), is a prerequisite assumption for the valid comparison of HRQoL scores across people from different subgroups (e.g., groups distinguished by gender, age, race. or health conditions). In general, DIF occurs when individuals from different groups respond differently to specific items in a questionnaire after controlling the construct being measured [1, 2]. The ordinal logistic regression (OLR) model is one of the well-known methods for the identification of DIF in psychometric research. The OLR model can evaluate both uniform and nonuniform DIF and can also control other categorical and continuous variables which may affect the results of DIF analysis [3, 4]. Uniform DIF occurs when the difference in item response probabilities remains constant across complete construct domains, whereas nonuniform DIF is evident when the direction of DIF differs across various parts of the construct scale [5, 6].

It is well documented that statistical inference based on the OLR model typically depends on the asymptotic properties of the maximum likelihood (ML) estimator [7]. When the sample size is relatively large, the sampling distribution of the ML estimators for the OLR coefficients is asymptotically normal and unbiased. However, when the sample size is very small, the robustness of the ML procedure and the asymptotic assumptions are violated. This in turn limits the use of the OLR model for DIF detection in small samples [4, 7]. Hence, it is essential to determine the extent to which the conventional OLR model is an efficient method for assessing DIF in the context of small sample sizes. A simulation study has shown that the OLR model needs sample sizes of about 200 for each group to obtain an adequate power and type I error rate in assessing DIF [8]. However, in HRQoL studies, researchers frequently encounter small sample sizes due to practical limitations such as prohibitive costs or low prevalence of a disease [9, 10].

Nowadays, a wide range of regularized or bias correction methods, known as machine learning approaches, have been proposed to overcome the problem of small sample size in binary and ordinal logistic regression models [7, 11–14]. In the binary logistic regression model, Firth's penalized maximum likelihood (PML) estimation approach was originally introduced to correct or reduce the small sample bias of the ML estimators [11]. The ML estimation method tends to produce overfitted models with a poor prediction performance in the presence of small or sparse samples, whereas the PML procedure offers some improvements by shrinking the regression coefficients. In reality, the PML method produces unbiased and finite estimates of regression coefficients for small sample situations in which ordinary ML estimation fails [11, 13, 15]. Recently, for ordinal response data, the elastic net regularized OLR model has been proposed by Wurm et al. to improve the estimation of the regression coefficients, as well as to perform variable selection [14]. The elastic net is another regularized regression technique that linearly combines the penalties used in the ridge and least absolute shrinkage and selection operator (LASSO) regression models [16]. The ridge and LASSO regressions are two well-known members of the regularization family of methods [12, 17]. The major difference between the two algorithms is that while the ridge method shrinks all of the regression coefficients to a nonzero value, the LASSO method shrinks some of the coefficients exactly to zero. The differences lie in the penalty terms used by each method. While the LASSO regression adds the sum of the absolute value of the magnitude of the regression coefficients to the ordinary likelihood function, the ridge regression adds the sum of the squared magnitude [12, 17].

A new psychometric study has shown that regularization techniques, as a special type of machine learning methods, outperform the conventional DIF detection procedures when the sample size is extremely small [18]. Although the effect of regularization methods on the performance of binary logistic regression models in detecting DIF has been evaluated by Lee in small sample size groups [5], such a statistical description has never been provided for the OLR model. For the binary logistic regression model, Lee has shown that when the sample size is relatively small, there is no difference between the conventional ML and PML estimation methods in terms of power and type I error rate for detecting DIF [5]. Hence, in the present study, we intended to propose a new application of the elastic net regularized OLR model, as a special type of machine learning approach, in assessing DIF when the sample sizes for one or both groups of interest are relatively small. Accordingly, by presenting a comprehensive simulation study, we investigated whether the statistical properties (power and type I error) of the OLR model for evaluating DIF, with and without applying regularized techniques, could be affected by the sample size, sample size ratio, DIF amount, scale length, and the value of the weighting parameter across the focal and reference groups. Furthermore, a real data set was also used to validate the simulation results.

## 2. Methods

The cumulative ordinal logistic regression (OLR) model, also known as the proportional odds model, was used to identify DIF across the two groups [4]. Moreover, the Monte Carlo simulation method was utilized to compare the statistical properties (power and type I error) of the regularized and nonregularized OLR models for detecting DIF.

### 2.1. DIF Detection Method Based on the Nonregularized OLR Model

The uniform and nonuniform DIF can be assessed by comparing three different nested OLR models as follows:

(1) $$\begin{array}{c} \text{Model }1:\text{Logit }\left[\text{P}\left(\text{Y}\leq \text{j}\right)\right]=\log\left(\frac{\text{P}\left(\text{Y}\leq \text{j}\right)}{1-\text{P}\left(\text{Y}\leq \text{j}\right)}\right)=\gamma_{0\text{j}}+\beta_{11}\theta \\ \text{Model }2:\text{Logit }\left[\text{P}\left(\text{Y}\leq \text{j}\right)\right]=\log\left(\frac{\text{P}\left(\text{Y}\leq \text{j}\right)}{1-\text{P}\left(\text{Y}\leq \text{j}\right)}\right)=\gamma_{0\text{j}}+\beta_{12}\theta +\beta_{22}\text{G}\\ \text{Model }3:\text{Logit }\left[\text{P}\left(\text{Y}\leq \text{j}\right)\right]=\log\left(\frac{\text{P}\left(\text{Y}\leq \text{j}\right)}{1-\text{P}\left(\text{Y}\leq \text{j}\right)}\right)=\gamma_{0\text{j}}+\beta_{13}\theta +\beta_{23}\text{G}+\beta_{33}\left(\theta \times \text{G}\right) \end{array}$$

Here, *P*(*Y* ≤ *j*) denotes the probability of choosing category *j* or below, *G* is a covariate indicating group membership with two levels (i.e., reference and focal groups), and *θ* is the observed ability of an examinee usually demonstrated by the total test score [3, 4].

The regression coefficients (*γ*s and *β*s) are unknown constants and are estimated by maximizing the log-likelihood (ML) function [7]. According to the above models, the presence of uniform DIF can be checked by testing whether the coefficient of group membership (*H*_0_ : *β*_22_ = 0) differs significantly from zero. This can be done by comparing the -2 log-likelihood values for models 1 and 2 with chi-square distribution with one degree of freedom. The interaction regression coefficient between the ability and group membership (*H*_0_ : *β*_33_ = 0) can be tested to evaluate nonuniform DIF by comparing the -2 log-likelihood values for models 2 and 3 with chi-square distribution with one degree of freedom [4]. It should be noted that since the DIF direction is different along the latent ability scale in nonuniform DIF, the nonuniform DIF items cancel each other out at the test level [19]. Therefore, the focus in this simulation study is on uniform DIF detection.

### 2.2. DIF Detection Method Based on the Elastic Net Regularized OLR Model

In this section, a new machine learning approach is proposed for DIF detection based on the elastic net regularized OLR model [14]. Detecting DIF through the regularized (elastic net) and nonregularized OLR models is exactly the same except that in the elastic net regularized OLR model, the uniform and nonuniform DIF can be detected by comparing the penalized log-likelihood values of models 1 and 2 and models 2 and 3, respectively [5].

The elastic net regularized OLR model is a linear combination of the LASSO and ridge regression models, and its penalized log-likelihood function can be formulated as follows:

(2) $$\begin{array}{c} l_{elastic\;net}=-\frac{1}{N}\times logL+\lambda \left[w\overset{p}{\underset{j=1}{\sum }}\left|\beta_{j}\right|+\frac{1}{2}\left(1-w\right)\overset{p}{\underset{j=1}{\sum }}\beta_{j}^{2}\right], \end{array}$$

in which *N* is the total sample size, log*L* denotes the conventional version of the log-likelihood function, and *λ* ≥ 0 and 0 ≤ *w* ≤ 1 are the regularization and weighting parameters, respectively [14, 16]. In this equation, *β*_*j*_ indicates the regression coefficients, and ∑_*j*=1_^*p*^*β*_*j*_^2^ (squared *L*_2_-norm of *β*_*j*_) and ∑_*j*=1_^*p*^|*β*_*j*_| (*L*_1_-norm of *β*_*j*_) represent the ridge and LASSO penalty terms, respectively [12, 17]. The elastic net OLR model is simplified to the LASSO OLR model when *w* = 1 and to the ridge OLR model when *w* = 0. It should also be noted that when *λ* = 0, the elastic net penalty term is eliminated, and the usual log-likelihood function of the ML method is obtained. The “ordinalNet” package in the R software was used to fit the elastic net regularized OLR model [14, 16].

In this simulation study, for any fixed value of *w* in the interval 0 ≤ *w* ≤ 1, the optimal value of *λ* was determined by the Bayesian information criterion (BIC). For the elastic net regularized OLR model, the BIC was calculated by the following equation:

(3) $$\begin{array}{c} \text{BIC}=-2\log L_{\lambda }+\log\left(N\right)\times N_{nonzero}, \end{array}$$

where log*L*_*λ*_ represents the log-likelihood value for the regression parameters estimated with regularization parameter *λ*, *N* is the total sample size, and *N*_nonzero_ indicates the estimated total number of nonzero parameters including the intercepts [14]. For a regular sequence of *λ* values, which is automatically generated by the “ordinalNet” package, the corresponding BIC values are calculated and sorted in a descending order according to their magnitude. Then, the package chooses the optimal value of *λ* in a way that the BIC value is minimized [14].

### 2.3. Generation of Simulated Data

In this study, Samejima's graded response model (GRM) [20], which is suitable for simulating items with ordered response categories, was used to produce five-category response data for measures with five or ten items. The mathematical equation for GRM is as follows:

(4) $$\begin{array}{c} P_{ij}\left(\theta \right)=P_{i}\left(Y\geq j\;|\;\theta \right)=\frac{1}{1+\exp\left(-a_{i}\left(\theta -b_{ij}\right)\right)}, \end{array}$$

where *P*_*ij*_(*θ*) is the probability of responding in or above category j of item *i*, *a*_*i*_ is the item discrimination parameter, *θ* is the latent trait (ability), and *b*_*ij*_ denotes the item difficulty (threshold) parameter for category *j* of item *i*. The item discrimination parameters were randomly generated from the uniform distribution over the interval (1, 2), and the difficulty and ability parameters were sampled from the standard normal distribution [1].

In the present simulation study, four factors including the total sample size (*N*), sample size ratio (*R*), scale length (*I*), and magnitude of uniform DIF that could influence the performance of the regularized and nonregularized OLR models were manipulated. Out of five or ten items in the scale, one item (item 1) was flagged with uniform DIF across the reference and focal groups. In order to simulate the moderate and severe uniform DIF, 0.4 and 0.8 were, respectively, added to the difficulty parameters (*b*_1*j*_) of item 1 in the focal group. Five sample sizes (*N* = 100, 150, 200, 300, and 400) and three levels of sample size ratio (*R* = 1, 2, and 3) were considered. When *N* = 100, for example, the conditions *n*_*r*_/*n*_*f*_ = 50/50, 67/33, and 75/25 were generated. In addition, two tests with 5 and 10 items (*I* = 5 and 10) were generated. Each item had five response categories (*J* = 5). The data were simulated using the “catIrt” package [21] as well as the “runif” and “rnorm” functions in the R statistical software (version: 4.0.2).

The main objective of this simulation study was to determine what the optimal value of the weighting parameter *w* should be to obtain the adequate power and type I error rate for the detection of DIF. In order to find the optimal value of *w*, the following sequence of weighting parameters was considered: *w* = 0, 0.01, 0.02, 0.03, 0.04, 0.05, 0.06, 0.07, 0.1, 0.5, and 1 (*w* = 0 and *w* = 1 corresponded to the ridge and LASSO regressions, respectively). For each *w*, the BIC was used to select the appropriate regularization parameter *λ* [14].

The power rate (true positive rate) is defined by the ratio of the times that the uniform DIF is rightly flagged by various methods across replications. Type I error rate (false-positive rate) also indicates the ratio of non-DIF items wrongly identified as having DIF in 1000 replications. They are averaged over all items without DIF [22].

## 3. Results

### 3.1. Uniform DIF Power and Type I Error Rates

Tables 1 and 2, respectively, show the power and type I error rates of the regularized (elastic net) and nonregularized OLR models to assess the presence of a moderate uniform DIF (DIF = 0.4) under different combinations of scale length (*I*), total sample size (*N*), sample size ratio (*R*), and response category number of five (*J* = 5).

According to Table 1, for *I* = 5 and regardless of *R*, the elastic net regularized OLR model with *w* = 0.1, as compared with the nonregularized OLR model, increased the power of detecting moderate uniform DIF (DIF = 0.4) approximately 35%, 21%, and 21.7% for *N* = 100, 150, and 200, respectively. In this case, the type I error rates of the regularized and nonregularized OLR models were close to the nominal level of 0.05 (Table 2). However, this amount of increase in power was approximately 15.5% and 12.6% for *N* = 300 and 400, respectively. In this situation, the average type I error rate of the nonregularized OLR model was close to the nominal level of 0.05, while it was approximately 0.07 for the elastic net regularized OLR model.

As demonstrated in Table 1, for *N* ≥ 300, *I* = 5, and *R* = 1, as compared with the nonregularized OLR model, increasing the weighting parameter (*w*) from 0.1 to 0.5 resulted in an increase of approximately 11.7% to 17.7% in the power of the elastic net regularized OLR model for detecting moderate uniform DIF (DIF = 0.4). In this case, the elastic net regularized OLR model tended to reach the adequate power of 80%, while its type I error rate was slightly above the nominal level of 0.05. However, for *I* = 10, the maximum power for detecting moderate uniform DIF (DIF = 0.4) was 0.587 and 0.462 for the LASSO and nonregularized OLR models, respectively.

Tables 3 and 4, respectively, represent the power and type I error rates of the regularized (elastic net) and nonregularized OLR models in detecting the presence of severe uniform DIF (DIF = 0.8) under different combinations of *I*, *N*, *R*, and *J* = 5. As shown in Table 3, when *I* = 5 and *R* = 1, the elastic net regularized OLR model with *w* = 0.05, as compared with the nonregularized OLR model, increased the power of detecting severe uniform DIF (DIF = 0.8) approximately 9% and 4.5% for *N* = 100 and 150, respectively. In this case, the type I error rates of the elastic net regularized and nonregularized OLR models were 0.058 and 0.078 for *N* = 100 and 150, respectively. Similar findings were also obtained for the elastic net regularized OLR model with *w* = 0.04 and 0.06. Hence, for a relatively small sample size (*N* ≤ 150), *I* = 5, and *R* = 1, the elastic net regularized OLR model with 0.04 ≤ *w* ≤ 0.06 achieved the adequate power of 80% and type I error rate of 0.05 for detecting severe uniform DIF (DIF = 0.8). However, for *N* ≥ 200, *I* = 5, and *R* = 1, the type I error rates were considerably higher than the nominal level of 0.05 for the elastic net regularized (*w* ≥ 0.03) and nonregularized OLR models. In the same conditions, the power of the elastic net regularized OLR model with 0 ≤ *w* ≤ 0.02 was equal to or greater than 80% in detecting severe uniform DIF (DIF = 0.8), while its type I error rate was below or close to the nominal level of 0.05. In addition, for *N* ≥ 200 and *I* = 5, similar findings were obtained when the sample size was unequal (*R* = 2 and 3) between the focal and reference groups.

Moreover, regardless of *R*, for the severe magnitude of DIF (DIF = 0.8), *I* = 10, and *N* ≥ 200, the powers of the regularized and nonregularized OLR models were close to or higher than 80%, and their type I error rates were below or close to the nominal level of 0.05.

On the other hand, for a severe magnitude of DIF (DIF = 0.8), *I* = 10, and a relatively small sample size (*N* ≤ 150), when the weighting parameter (*w*) increased from 0.03 to 1, the power of the elastic net regularized OLR model, as compared with the nonregularized OLR model, increased from 11.2% to 24.8%. In this case, the regularized and nonregularized OLR models preserved the type I error rates approximately close to the nominal level of 0.05.

**Figure 1:**
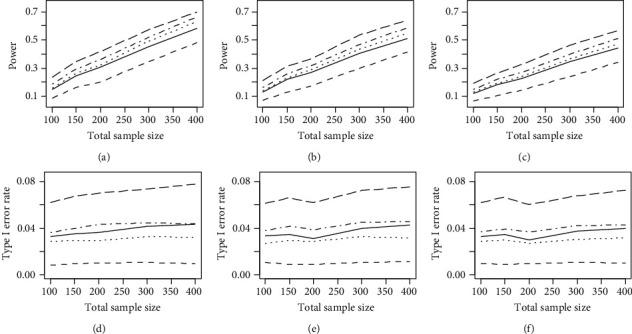
The average powers and type I error rates of the nonregularized OLR model (solid lines), elastic net OLR model with *w* = 0 (ridge) (dashed lines), elastic net OLR model with *w* = 0.03 (dotted lines), elastic net OLR model with *w* = 0.05 (dot-dashed lines), and elastic net OLR model with *w* = 1 (LASSO) (long-dashed lines) on measures with five and ten items for moderate DIF (DIF = 0.4). (a) DIF = 0.4 and *n*_*r*_ = *n*_*f*_. (b) DIF = 0.4 and *n*_*r*_ = 2*n*_*f*_. (c) DIF = 0.4 and *n*_*r*_ = 3*n*_*f*_. (d) DIF = 0.4 and *n*_*r*_ = *n*_*f*_. (e) DIF = 0.4 and *n*_*r*_ = 2*n*_*f*_. (f) DIF = 0.4 and *n*_*r*_ = 3*n*_*f*_.

**Figure 2:**
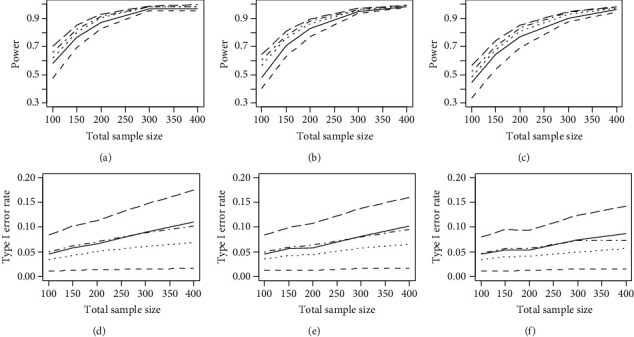
The average powers and type I error rates of the nonregularized OLR (solid lines), elastic net OLR model with *w* = 0 (ridge) (dashed lines), elastic net OLR model with *w* = 0.03 (dotted lines), elastic net OLR model with *w* = 0.05 (dot-dashed lines), and elastic net OLR model with *w* = 1 (LASSO) (long-dashed lines) on measures with five and ten items for severe DIF (DIF = 0.8). (a) DIF = 0.8 and *n*_*r*_ = *n*_*f*_. (b) DIF = 0.8 and *n*_*r*_ = 2*n*_*f*_. (c) DIF = 0.8 and *n*_*r*_ = 3*n*_*f*_. (d) DIF = 0.8 and *n*_*r*_ = *n*_*f*_. (e) DIF = 0.8 and *n*_*r*_ = 2*n*_*f*_. (f) DIF = 0.8 and *n*_*r*_ = 3*n*_*f*_.

Figures 1 and 2 demonstrate the average powers and type I error rates of the regularized and nonregularized OLR models on measures with five and ten items for moderate and severe DIF, respectively. According to these figures, regardless of DIF magnitude, sample size ratio, and the number of items, the elastic net regularized OLR model with *w* = 0 (ridge) and *w* = 1 (LASSO) had the lowest and highest power for detecting uniform DIF, respectively. Moreover, the nonregularized OLR model and the elastic net regularized OLR model with *w* = 0.03 had an approximately equal power. Additionally, as shown in Figures 1 and 2, the average type I error rate was generally below or close to the nominal level of 0.05 for the elastic net regularized (*w* = 0, 0.03, and 0.05) and nonregularized OLR models (range: 0.029-0.069). However, it was above the nominal level of 0.05 for the elastic net regularized OLR model with *w* = 1 (range: 0.073-0.127).

### 3.2. Real Data Analysis

In the present section, a real data set was employed to validate the simulation results. The data set was composed of 72 children and adolescents with attention-deficit/hyperactivity disorder (ADHD) (aged 8-18; 81.9% females; 18.1% males) who had referred with their parents to the Child and Adolescent Psychiatry Clinics affiliated with Shiraz University of Medical Sciences, Shiraz, Iran. The children and their parents, respectively, completed the child self-reports and the proxy reports of the Persian version of the PedsQL™ 4.0 Generic Core Scales [23]. The 23-item PedsQL™ 4.0 questionnaire consists of 4 subscales: physical functioning (8 items), social functioning (5 items), emotional functioning (5 items), and school functioning (5 items). A 5-point Likert scale from 0 (never) to 4 (almost always) is used to measure the respondents' perception on each item.

The results of the DIF analysis of the PedsQL™ 4.0 instrument across children with ADHD and their parents based on the regularized and nonregularized OLR models are summarized in Table 5. Our results showed that while the elastic net regularized OLR model with *w* ≥ 0.03 and the nonregularized OLR model exhibited seven out of the 23 items with uniform DIF, the elastic net regularized OLR model with *w* = 0, 0.01, and 0.02 identified four, four, and six items with uniform DIF, respectively. In addition, according to Table 5, increasing the value of the weighting parameter (*w*) from 0 to 1 resulted in an increase in the power of the elastic net regularized OLR model for detecting uniform DIF. These results were consistent with the simulation findings in Tables 1 and 3, especially when the sample size was extremely small (*N* ≤ 150).

## 4. Discussion

A small sample size is a challenging issue in assessing DIF for ordinal response data. To the best of our knowledge, this is the first study that introduces a new application of the elastic net regularized OLR model, as a special type of machine learning method, for detecting DIF in small sample size groups. One of the main advantages of the elastic net regularized OLR method over other regularization methods for DIF detection is that it provides the researcher with a wide range of models by varying the weighting parameter (*w*) over the interval from 0 to 1. However, choosing an optimal value for *w* is the key issue in using elastic net regularized models [14, 16]. The present simulation study demonstrated that the elastic net regularized OLR model with 0.03 ≤ *w* ≤ 0.06 generally outperformed the nonregularized OLR model in terms of the statistical power to identify DIF especially when the sample size was extremely small (*N* ≤ 150), the number of items was 10 (*I* = 10), and DIF was severe (DIF = 0.8). These findings were different from those of Lee who showed that the nonregularized binary logistic regression model performed slightly better than the regularized logistic regression model (based on the PML estimation method) in detecting DIF for small samples [5].

The current study also showed that when *w* was greater than 0.1 (*w* = 0.5 and 1), the value of the regularization parameter (*λ*) converged to zero, indicating that the likelihood function of the elastic net regularized OLR model was reduced to the likelihood function of the nonregularized OLR model. This can lead to almost similar results for the elastic net regularized (*w* > 0.1) and nonregularized OLR models in identifying DIF.

Although all previous studies have used the LASSO regularized method for DIF assessment with binary-scored items [18, 24–26], the findings of the current study showed that we should be cautious about using the LASSO regularized OLR model (*w* = 1) for DIF analysis. This is because when the number of items is five (*I* = 5), the LASSO regularized OLR model inflates the type I error rate to 0.2. However, for larger scales (*I* = 10) and when the sample size is relatively small (*N* ≤ 150), using the LASSO regularized OLR model for the identification of severe uniform DIF (DIF = 0.8) is strongly recommended. This finding also confirmed the results of a previous simulation study which showed that the regularized logistic regression model with LASSO penalty outperformed the conventional logistic regression model in DIF detection when the sample size was small and the number of items was 20 (*I* = 20) [24].

A further novel finding of the present study is that the elastic net regularized OLR model with 0.03 ≤ *w* ≤ 0.07 becomes necessary for DIF analysis when the scale length and sample size are relatively small (*I* = 5 and *N* ≤ 150). These findings are different from those in the previous study conducted by Scott et al. where the nonregularized OLR model was used to detect DIF when the sample size was higher than or equal to 200 (*N* ≥ 200) [8]. They simulated subscales with 2, 3, 4, 5, 10, and 20 items and showed that the effect of the number of items in the scale was relatively small on the results of DIF detection based on the nonregularized OLR model [8]. Accordingly, further simulation studies are needed to explore the effect of varying the length of the scale on the performance of the LASSO OLR model for DIF analysis.

Furthermore, the findings of the current study revealed that when *N* ≤ 150 and DIF was moderate (DIF = 0.4), the ridge regularized OLR model (*w* = 0) had the lowest power in detecting DIF as compared with the other regularized OLR model and the nonregularized OLR models. In these conditions, we should be cautious about using the ridge regularized OLR model (*w* = 0) for detecting uniform DIF because it can lead to false negative results.

On the other hand, comparing the regularized and nonregularized OLR models for the real ADHD data set confirmed the results of the present simulation for detecting DIF. The current simulation study demonstrated that by increasing the value of the weighting parameter (*w*), the power of the elastic net regularized OLR model will be increased. Accordingly, in the real data set, the elastic net regularized OLR model with *w* equal to or greater than 0.03 was more sensitive in detecting uniform DIF across children with ADHD and their parents than the elastic net regularized OLR model with 0 ≤ *w* ≤ 0.02.

One of the interesting findings of the current study was that when *N* ≥ 200 and DIF = 0.8 (severe DIF), the regularized and nonregularized OLR models had an adequate power for detecting DIF. These findings were similar to those of Magis et al. who reported that when the sample size was large, the regularized LASSO logistic regression and the nonregularized logistic regression models yielded similar results for identifying DIF in binary response data [24]. However, the current simulation study revealed that when the magnitude of DIF was severe (DIF = 0.8), *N* ≥ 200, and the number of items was five (*I* = 5), the elastic net regularized OLR model with *w* ≥ 0.03 and the nonregularized OLR model inflated type I error rate up to the unacceptable level of 26.6%.

In the present study, we simulated data based on moderate (DIF = 0.4) and severe (DIF = 0.8) DIF conditions. Similar to our study, Hidalgo et al. manipulated two levels of differences in threshold parameters (0.4 and 0.8) to simulate moderate and large magnitudes of DIF [27]. Moreover, according to Li and Zumbo, the magnitude of DIF equal to 0.4, 0.6, and 0.8 can be considered small, moderate, and large DIF, respectively [28]. However, Scott et al. considered three levels of DIF magnitude including 0.2, 0.5, and 1 to generate items with small, moderate, and large DIF, respectively [8]. Although there is no consensus on the definition of DIF magnitude, it seems that the magnitude of DIF from 0.4 to 1 is a feasible option to simulate items with moderate and severe DIF.

Although various criteria including the Bayesian information criterion (BIC) and cross-validation method can be used to obtain the optimal tuning parameter *λ* [14, 16, 18], in the present research, the BIC was only used to assess DIF. In DIF analysis, the cross-validation and BIC have technical and theoretical differences. The BIC has been designed for variable selection, whereas cross-validation is generally used to select the optimal model for prediction [26]. Moreover, previous studies have demonstrated that the cross-validation method tends to have higher false-positive rates in detecting DIF than the BIC [18, 25]. Because variable selection is much more important in DIF assessment than prediction, the findings of the present study were interpreted based on the BIC [26].

This research had some limitations which are as follows. Although our simulation study was restricted to identifying only the uniform DIF, the elastic net regularized OLR model could also be extended to cover both uniform and nonuniform DIF. In addition, the regularized model used in the present research contains a maximum of two predictors which do not appear to be collinear either. Hence, in the future study, the performance of the elastic net regularized OLR model for DIF analysis should be assessed when several highly correlated continuous and categorical covariates are included in the model [14]. Finally, it should be noted that traditional DIF detection approaches are very sensitive to missing item responses and the questionnaires with missing data are usually excluded from the DIF analysis [29]. Hence, in the present study, we did not simulate items with missing data. Accordingly, as a special type of machine learning method, the elastic net regularized OLR model could be a viable choice for identifying DIF with missing data and without needing imputation [24].

## 5. Conclusion

Technically, the findings of the present study confirmed the idea proposed by Belzak and Bauer where regularization methods, as a special type of machine learning technique, could compensate for the limitation of the conventional DIF detection methods when the sample size is relatively small [18]. This study provided a guideline for researchers who conduct DIF studies with extremely small sample sizes. In general, for extremely small sample sizes (*N* ≤ 150), the elastic net regularized OLR model with 0.03 ≤ *w* ≤ 0.1 outperformed the nonregularized OLR model in terms of power and type I error rate. Moreover, in future studies, the advantages of the elastic net regularized OLR model in dealing with missing data and collinearity problem in the context of DIF analysis should be assessed.

## Figures and Tables

**Table 1:** The powers of the regularized (elastic net) and non-regularized OLR models in detecting moderate uniform DIF (DIF=0.4) when J=5.

I	Ratio	N	OLR	Ridge	Elastic net OLR									LASSO
				*w*=0	*w*=0.01	*w*=0.02	*w*=0.03	*w*=0.04	*w*=0.05	*w*=0.06	*w*=0.07	*w*=0.1	*w*=0.5	*w*=1
5	n_r_=n_f_	100	0.179	0.100	0.098	0.146	0.175	0.192	0.215	0.224	0.233	0.247	0.277	0.281
		150	0.317	0.184	0.177	0.265	0.310	0.332	0.347	0.357	0.361	0.370	0.412	0.413
		200	0.365	0.217	0.210	0.190	0.360	0.389	0.400	0.409	0.418	0.433	0.479	0.483
		300	0.559	0.409	0.392	0.518	0.572	0.594	0.610	0.621	0.625	0.632	0.673	0.677
		400	0.702	0.550	0.528	0.673	0.734	0.754	0.770	0.773	0.772	0.774	0.808	0.811

5	n_r_=2n_f_	100	0.161	0.068	0.065	0.126	0.155	0.174	0.188	0.199	0.207	0.215	0.257	0.260
		150	0.279	0.140	0.129	0.230	0.266	0.291	0.300	0.309	0.320	0.333	0.366	0.366
		200	0.334	0.204	0.196	0.287	0.333	0.360	0.375	0.383	0.397	0.409	0.439	0.441
		300	0.497	0.344	0.329	0.452	0.498	0.527	0.544	0.556	0.562	0.579	0.626	0.632
		400	0.629	0.499	0.474	0.598	0.644	0.661	0.683	0.694	0.700	0.708	0.737	0.739

5	n_r_=3n_f_	100	0.143	0.067	0.064	0.108	0.135	0.149	0.158	0.167	0.175	0.190	0.221	0.225
		150	0.219	0.108	0.103	0.176	0.211	0.232	0.244	0.249	0.257	0.278	0.306	0.308
		200	0.279	0.171	0.160	0.250	0.280	0.300	0.314	0.322	0.331	0.346	0.379	0.379
		300	0.430	0.279	0.265	0.373	0.432	0.457	0.472	0.481	0.486	0.503	0.543	0.546
		400	0.539	0.397	0.381	0.507	0.556	0.583	0.596	0.604	0.610	0.619	0.652	0.655

*λ*_BIC_∗			-	0.380	0.381	0.190	0.130	0.095	0.076	0.063	0.054	0.038	0.008	0.004

10	n_r_=n_f_	100	0.117	0.075	0.072	0.116	0.143	0.153	0.161	0.163	0.166	0.171	0.189	0.190
		150	0.173	0.138	0.133	0.184	0.216	0.232	0.235	0.240	0.245	0.256	0.272	0.277
		200	0.248	0.189	0.183	0.262	0.285	0.305	0.318	0.324	0.333	0.343	0.355	0.358
		300	0.350	0.283	0.276	0.360	0.405	0.424	0.432	0.440	0.442	0.452	0.472	0.473
		400	0.462	0.410	0.394	0.502	0.531	0.548	0.558	0.562	0.563	0.565	0.587	0.587

10	n_r_=2n_f_	100	0.102	0.072	0.070	0.112	0.123	0.135	0.142	0.145	0.147	0.156	0.165	0.166
		150	0.167	0.121	0.120	0.172	0.198	0.211	0.222	0.228	0.232	0.238	0.258	0.258
		200	0.207	0.144	0.142	0.218	0.241	0.250	0.259	0.263	0.267	0.275	0.293	0.293
		300	0.314	0.256	0.242	0.332	0.364	0.380	0.394	0.401	0.403	0.410	0.432	0.434
		400	0.389	0.333	0.324	0.417	0.456	0.479	0.487	0.492	0.499	0.511	0.537	0.537

10	n_r_=3n_f_	100	0.099	0.064	0.062	0.098	0.119	0.133	0.141	0.146	0.148	0.150	0.158	0.159
		150	0.146	0.098	0.096	0.150	0.175	0.188	0.194	0.199	0.203	0.211	0.220	0.222
		200	0.168	0.114	0.110	0.165	0.200	0.220	0.224	0.229	0.234	0.245	0.274	0.276
		300	0.264	0.204	0.196	0.272	0.300	0.318	0.330	0.336	0.345	0.354	0.375	0.376
		400	0.349	0.281	0.265	0.367	0.390	0.411	0.426	0.433	0.441	0.459	0.482	0.483

*λ*_BIC_∗			-	0.315	0.315	0.160	0.105	0.080	0.063	0.052	0.045	0.032	0.006	0.003

*Note*: DIF: differential item functioning; I: number of items in the scale; J: number of response categories; LASSO: least absolute shrinkage and selection operator; *λ*: regularization parameter; OLR: ordinal logistic regression; *w*: weighting parameter; Ratio: sample size ratio between the focal and reference groups; n_f_ and n_r_ indicate the sample sizes in the focal and reference groups, respectively; N: the total sample size (N=n_f_ +n_r_). ^∗^These *λ* values were obtained according to the Bayesian information criterion (BIC).

**Table 2:** The type I error rates of the regularized (elastic net) and non-regularized OLR models in detecting moderate uniform DIF (DIF=0.4) when J=5.

I	Ratio	N	OLR	Ridge	Elastic net OLR									LASSO
				*w*=0	*w*=0.01	*w*=0.02	*w*=0.03	*w*=0.04	*w*=0.05	*w*=0.06	*w*=0.07	*w*=0.1	*w*=0.5	*w*=1
5	n_r_=n_f_	100	0.037	0.007	0.007	0.020	0.028	0.033	0.036	0.040	0.042	0.048	0.066	0.068
		150	0.040	0.009	0.008	0.021	0.029	0.038	0.042	0.046	0.050	0.056	0.075	0.076
		200	0.042	0.008	0.007	0.018	0.028	0.038	0.046	0.050	0.056	0.063	0.080	0.081
		300	0.052	0.011	0.010	0.023	0.038	0.045	0.051	0.057	0.060	0.070	0.088	0.089
		400	0.056	0.009	0.008	0.023	0.037	0.046	0.053	0.061	0.066	0.077	0.098	0.100

5	n_r_=2n_f_	100	0.038	0.012	0.011	0.018	0.027	0.034	0.040	0.042	0.044	0.051	0.067	0.068
		150	0.038	0.006	0.006	0.018	0.026	0.034	0.040	0.045	0.049	0.058	0.071	0.073
		200	0.035	0.008	0.006	0.020	0.030	0.035	0.040	0.043	0.046	0.054	0.067	0.067
		300	0.053	0.010	0.010	0.029	0.037	0.045	0.053	0.059	0.061	0.070	0.087	0.089
		400	0.054	0.012	0.010	0.021	0.034	0.046	0.052	0.056	0.062	0.070	0.091	0.093

5	n_r_=3n_f_	100	0.036	0.009	0.009	0.019	0.029	0.035	0.039	0.044	0.046	0.052	0.069	0.071
		150	0.039	0.008	0.007	0.018	0.030	0.034	0.040	0.048	0.051	0.058	0.072	0.074
		200	0.036	0.010	0.008	0.021	0.030	0.037	0.041	0.046	0.050	0.055	0.069	0.070
		300	0.041	0.010	0.009	0.020	0.030	0.038	0.044	0.048	0.053	0.061	0.075	0.077
		400	0.052	0.011	0.008	0.025	0.036	0.043	0.049	0.055	0.059	0.068	0.088	0.090

*λ*_BIC_∗			-	0.380	0.381	0.190	0.130	0.095	0.076	0.063	0.054	0.038	0.008	0.004

10	n_r_=n_f_	100	0.029	0.009	0.008	0.021	0.029	0.034	0.037	0.040	0.041	0.046	0.056	0.056
		150	0.030	0.010	0.009	0.020	0.030	0.035	0.038	0.042	0.045	0.050	0.058	0.059
		200	0.030	0.012	0.010	0.022	0.030	0.036	0.040	0.043	0.045	0.050	0.058	0.059
		300	0.031	0.010	0.008	0.022	0.028	0.034	0.038	0.041	0.044	0.048	0.058	0.059
		400	0.031	0.010	0.009	0.020	0.027	0.032	0.035	0.040	0.041	0.046	0.055	0.056

10	n_r_=2n_f_	100	0.029	0.009	0.009	0.020	0.027	0.031	0.036	0.038	0.040	0.045	0.055	0.055
		150	0.031	0.012	0.011	0.022	0.032	0.038	0.043	0.045	0.047	0.050	0.059	0.059
		200	0.027	0.009	0.008	0.019	0.027	0.034	0.037	0.040	0.042	0.046	0.056	0.057
		300	0.027	0.011	0.009	0.020	0.028	0.033	0.037	0.039	0.041	0.046	0.055	0.056
		400	0.031	0.010	0.009	0.021	0.029	0.035	0.039	0.041	0.043	0.047	0.057	0.058

10	n_r_=3n_f_	100	0.029	0.010	0.009	0.021	0.028	0.031	0.035	0.037	0.040	0.044	0.053	0.053
		150	0.030	0.009	0.009	0.022	0.030	0.034	0.038	0.041	0.043	0.048	0.058	0.059
		200	0.024	0.009	0.008	0.017	0.024	0.030	0.033	0.036	0.038	0.042	0.050	0.051
		300	0.034	0.011	0.010	0.023	0.031	0.036	0.040	0.043	0.045	0.049	0.058	0.059
		400	0.028	0.009	0.008	0.021	0.027	0.032	0.036	0.038	0.041	0.045	0.054	0.055

*λ*_BIC_∗			-	0.315	0.315	0.160	0.105	0.080	0.063	0.052	0.045	0.032	0.006	0.003

*Note*: DIF: differential item functioning; I: number of items in the scale; J: number of response categories; LASSO: least absolute shrinkage and selection operator; OLR: ordinal logistic regression; *w*: weighting parameter; Ratio: sample size ratio between the focal and reference groups; n_f_ and n_r_ indicate sample sizes in the focal and reference groups, respectively; N: total sample size (N=n_f_ +n_r_). ^∗^These *λ* values were obtained according to the Bayesian information criterion (BIC).

**Table 3:** The powers of the regularized (elastic net) and non-regularized OLR models in detecting severe uniform DIF (DIF=0.8) when J=5.

I	Ratio	N	OLR	Ridge	Elastic-net OLR									LASSO
				*w*=0	*w*=0.01	*w*=0.02	*w*=0.03	*w*=0.04	*w*=0.05	*w*=0.06	*w*=0.07	*w*=0.1	*w*=0.5	*w*=1
5	n_r_=n_f_	100	0.705	0.564	0.550	0.679	0.727	0.754	0.767	0.774	0.778	0.790	0.808	0.809
		150	0.867	0.789	0.781	0.860	0.889	0.901	0.906	0.910	0.914	0.917	0.931	0.932
		200	0.940	0.894	0.885	0.944	0.958	0.964	0.966	0.968	0.969	0.969	0.971	0.971
		300	0.995	0.985	0.984	0.996	0.997	0.997	0.997	0.997	0.997	0.997	0.997	0.997
		400	0.998	1.000	1.000	1.000	1.000	1.000	1.000	1.000	1.000	1.000	1.000	1.000

5	n_r_=2n_f_	100	0.622	0.471	0.464	0.600	0.646	0.673	0.691	0.701	0.703	0.717	0.738	0.744
		150	0.811	0.733	0.729	0.817	0.851	0.862	0.871	0.873	0.879	0.886	0.898	0.899
		200	0.912	0.850	0.845	0.920	0.931	0.940	0.947	0.951	0.951	0.953	0.961	0.961
		300	0.989	0.987	0.975	0.986	0.989	0.990	0.991	0.992	0.992	0.993	0.995	0.995
		400	0.999	0.997	0.997	0.999	1.000	1.000	1.000	1.000	1.000	1.000	1.000	1.000

5	n_r_=3n_f_	100	0.557	0.400	0.393	0.519	0.567	0.559	0.613	0.623	0.631	0.648	0.668	0.670
		150	0.747	0.620	0.610	0.737	0.770	0.784	0.794	0.802	0.807	0.815	0.840	0.841
		200	0.873	0.785	0.777	0.862	0.892	0.907	0.913	0.915	0.918	0.918	0.931	0.932
		300	0.968	0.950	0.947	0.970	0.978	0.981	0.983	0.982	0.982	0.984	0.988	0.988
		400	0.995	0.985	0.984	0.995	0.995	0.996	0.996	0.997	0.997	0.997	0.997	0.997

*λ*_BIC_∗			-	0.380	0.380	0.190	0.130	0.095	0.076	0.063	0.054	0.038	0.008	0.004

10	n_r_=n_f_	100	0.456	0.383	0.377	0.486	0.518	0.543	0.548	0.554	0.559	0.576	0.596	0.597
		150	0.665	0.592	0.580	0.687	0.713	0.726	0.737	0.746	0.749	0.760	0.773	0.774
		200	0.800	0.763	0.754	0.835	0.855	0.860	0.861	0.864	0.868	0.872	0.888	0.888
		300	0.940	0.921	0.913	0.951	0.963	0.967	0.967	0.966	0.967	0.968	0.976	0.976
		400	0.979	0.971	0.968	0.988	0.990	0.992	0.991	0.991	0.991	0.991	0.993	0.993

10	n_r_=2n_f_	100	0.341	0.336	0.331	0.433	0.485	0.503	0.518	0.523	0.522	0.534	0.545	0.547
		150	0.606	0.530	0.521	0.619	0.665	0.674	0.689	0.698	0.703	0.712	0.719	0.719
		200	0.748	0.687	0.676	0.770	0.796	0.809	0.813	0.814	0.820	0.827	0.832	0.832
		300	0.907	0.879	0.870	0.916	0.929	0.933	0.935	0.937	0.940	0.947	0.950	0.950
		400	0.965	0.958	0.955	0.973	0.978	0.979	0.981	0.981	0.982	0.982	0.987	0.987

10	n_r_=3n_f_	100	0.341	0.274	0.263	0.361	0.400	0.420	0.432	0.437	0.441	0.447	0.464	0.464
		150	0.545	0.459	0.450	0.558	0.591	0.605	0.612	0.623	0.626	0.635	0.643	0.644
		200	0.667	0.596	0.589	0.678	0.721	0.737	0.749	0.757	0.751	0.761	0.771	0.771
		300	0.835	0.804	0.795	0.857	0.882	0.895	0.900	0.902	0.905	0.909	0.913	0.913
		400	0.935	0.905	0.896	0.941	0.951	0.958	0.960	0.960	0.960	0.960	0.963	0.964

*λ*_BIC_∗			-	0.315	0.315	0.160	0.105	0.080	0.063	0.052	0.045	0.032	0.006	0.003

*Note*: DIF: differential item functioning; I: number of items in the scale; J: number of response categories; LASSO: least absolute shrinkage and selection operator; *λ*: regularization parameter; OLR: ordinal logistic regression; *w*: weighting parameter; Ratio: sample size ratio between the focal and reference groups; n_f_ and n_r_ indicate sample sizes in the focal and reference groups, respectively; N: total sample size (N=n_f_ +n_r_). ^∗^These *λ* values were obtained according to the Bayesian information criterion (BIC).

**Table 4:** The type I error rates of the regularized (elastic net) and non-regularized OLR models in detecting severe uniform DIF (DIF=0.8) when J=5.

I	Ratio	N	OLR	Ridge	Elastic-net OLR									LASSO
				*w*=0	*w*=0.01	*w*=0.02	*w*=0.03	*w*=0.04	*w*=0.05	*w*=0.06	*w*=0.07	*w*=0.1	*w*=0.5	*w*=1
5	n_r_=n_f_	100	0.058	0.012	0.010	0.027	0.038	0.048	0.058	0.066	0.075	0.084	0.104	0.106
		150	0.078	0.014	0.013	0.034	0.051	0.065	0.078	0.086	0.094	0.108	0.132	0.134
		200	0.094	0.013	0.011	0.038	0.065	0.080	0.089	0.098	0.106	0.121	0.149	0.150
		300	0.135	0.018	0.017	0.054	0.082	0.108	0.124	0.137	0.147	0.166	0.209	0.213
		400	0.172	0.021	0.018	0.061	0.099	0.128	0.150	0.167	0.181	0.206	0.261	0.266

5	n_r_=2n_f_	100	0.059	0.015	0.014	0.030	0.042	0.053	0.059	0.067	0.072	0.081	0.105	0.107
		150	0.076	0.012	0.012	0.030	0.049	0.064	0.072	0.081	0.088	0.102	0.127	0.128
		200	0.080	0.014	0.012	0.035	0.055	0.071	0.081	0.091	0.099	0.112	0.143	0.145
		300	0.121	0.022	0.020	0.052	0.080	0.096	0.111	0.120	0.131	0.151	0.194	0.197
		400	0.155	0.021	0.018	0.056	0.089	0.112	0.135	0.151	0.162	0.185	0.232	0.234

5	n_r_=3n_f_	100	0.059	0.012	0.012	0.026	0.039	0.048	0.056	0.063	0.068	0.076	0.099	0.102
		150	0.072	0.011	0.009	0.032	0.047	0.062	0.070	0.078	0.084	0.098	0.122	0.124
		200	0.077	0.015	0.014	0.033	0.053	0.065	0.074	0.082	0.087	0.101	0.125	0.126
		300	0.103	0.017	0.015	0.042	0.062	0.081	0.096	0.108	0.118	0.137	0.169	0.171
		400	0.131	0.018	0.016	0.051	0.078	0.099	0.096	0.131	0.143	0.166	0.202	0.206

*λ*_BIC_∗			-	0.380	0.380	0.190	0.130	0.095	0.076	0.063	0.054	0.038	0.008	0.004

10	n_r_=n_f_	100	0.032	0.011	0.009	0.023	0.032	0.037	0.041	0.044	0.048	0.052	0.061	0.062
		150	0.037	0.010	0.010	0.025	0.034	0.042	0.047	0.050	0.053	0.058	0.069	0.070
		200	0.039	0.014	0.012	0.027	0.037	0.045	0.050	0.054	0.057	0.062	0.074	0.075
		300	0.044	0.013	0.011	0.027	0.039	0.047	0.053	0.057	0.060	0.067	0.078	0.079
		400	0.047	0.012	0.010	0.026	0.039	0.048	0.053	0.059	0.063	0.070	0.083	0.085

10	n_r_=2n_f_	100	0.033	0.010	0.009	0.023	0.030	0.035	0.040	0.043	0.045	0.050	0.060	0.061
		150	0.038	0.012	0.011	0.026	0.035	0.041	0.045	0.050	0.052	0.059	0.069	0.069
		200	0.036	0.010	0.009	0.024	0.034	0.041	0.045	0.049	0.051	0.057	0.068	0.069
		300	0.041	0.011	0.010	0.025	0.035	0.044	0.049	0.054	0.058	0.064	0.076	0.077
		400	0.049	0.012	0.011	0.027	0.040	0.048	0.054	0.058	0.063	0.070	0.085	0.086

10	n_r_=3n_f_	100	0.031	0.011	0.010	0.023	0.029	0.034	0.038	0.041	0.043	0.048	0.058	0.058
		150	0.035	0.010	0.009	0.024	0.033	0.039	0.044	0.048	0.051	0.056	0.065	0.066
		200	0.031	0.009	0.009	0.020	0.029	0.035	0.040	0.044	0.046	0.050	0.060	0.061
		300	0.045	0.013	0.012	0.028	0.038	0.047	0.050	0.054	0.058	0.064	0.077	0.078
		400	0.042	0.011	0.009	0.024	0.035	0.045	0.050	0.054	0.058	0.066	0.078	0.078

*λ*_BIC_∗			-	0.315	0.315	0.160	0.105	0.080	0.063	0.052	0.045	0.032	0.006	0.003

*Note*: DIF: differential item functioning; I: number of items in the scale; J: number of response categories; LASSO: least absolute shrinkage and selection operator; OLR: ordinal logistic regression; *w*: weighting parameter; Ratio: sample size ratio between the focal and reference groups; n_f_ and n_r_ indicate sample sizes in the focal and reference groups, respectively; N: total sample size (N=n_f_ +n_r_). ^∗^These *λ* values were obtained according to the Bayesian information criterion (BIC).

**Table 5:** The results of the DIF analysis for the PedsQL^TM^ 4.0 across children with ADHD and their parents based on the regularized (elastic net) and non-regularized OLR models.

	OLR	Ridge	Elastic net OLR									LASSO
		*w*=0	*w*=0.01	*w*=0.02	*w*=0.03	*w*=0.04	*w*=0.05	*w*=0.06	*w*=0.07	*w*=0.1	*w*=0.5	*w*=1
***Physical Functioning***	*P*-value											

1. Hard to walk more than a block	0.453	1.000	1.000	1.000	1.000	0.791	0.714	0.663	0.628	0.566	0.467	0.459
2. Hard to run	0.553	1.000	1.000	1.000	1.000	0.838	0.772	0.729	0.699	0.649	0.567	0.560
3. Hard to do sports or exercises	0.929	0.543	0.551	0.723	0.844	1.000	1.000	1.000	1.000	1.000	1.000	1.000
4. Hard to lift something heavy	**0.007**	0.146	0.154	0.059	**0.035**	**0.024**	**0.019**	**0.016**	**0.014**	**0.011**	**0.007**	**0.007**
5. Hard to take a bath or shower	**0.001**	**0.006**	**0.007**	**0.003**	**0.002**	**0.002**	**0.001**	**0.001**	**0.001**	**0.001**	**0.001**	**0.001**
6. Hard to do chores around house	0.070	0.276	0.290	0.169	0.130	0.108	0.098	0.091	0.087	0.080	0.071	0.070
7. Hurt or ache	0.572	0.442	0.452	0.494	0.519	0.536	0.546	0.552	0.557	0.564	0.575	0.575
8. Low energy	0.129	0.114	0.118	0.112	0.114	0.118	0.120	0.122	0.123	0.126	0.129	0.129

***Emotional Functioning***												

1. Feel afraid or scared	0.160	0.514	0.537	0.367	0.299	0.257	0.234	0.218	0.208	0.189	0.163	0.161
2. Feel sad or blue	**0.012**	**0.037**	**0.039**	**0.023**	**0.019**	**0.017**	**0.015**	**0.015**	**0.014**	**0.013**	**0.012**	**0.012**
3. Feel angry	**0.003**	**0.014**	**0.014**	**0.007**	**0.005**	**0.004**	**0.004**	**0.004**	**0.003**	**0.003**	**0.003**	**0.003**
4. Trouble sleeping	0.909	0.820	0.823	1.000	1.000	1.000	1.000	1.000	1.000	1.000	1.000	0.963
5. Worry about what will happen	**0.003**	0.074	0.078	**0.027**	**0.015**	**0.010**	**0.008**	**0.006**	**0.006**	**0.004**	**0.003**	**0.003**

***Social Functioning***												

1. Trouble getting along with peers	**0.024**	0.074	0.077	**0.047**	**0.038**	**0.033**	**0.031**	**0.029**	**0.028**	**0.026**	**0.024**	**0.024**
2. Other kids not wanting to be friends	0.985	0.925	0.928	1.000	1.000	1.000	1.000	1.000	1.000	1.000	1.000	1.000
3. Teased	0.167	0.298	0.309	0.250	0.226	0.210	0.201	0.195	0.190	0.182	0.169	0.168
4. Doing things other peers do	0.226	0.442	0.459	0.357	0.316	0.289	0.275	0.264	0.258	0.246	0.229	0.227
5. Hard to keep up when play with others	**0.002**	**0.041**	**0.043**	**0.016**	**0.010**	**0.007**	**0.005**	**0.005**	**0.004**	**0.003**	**0.002**	**0.002**

***School Functioning***												

1. Hard to concentrate	0.576	0.644	0.648	1.000	1.000	1.000	1.000	1.000	1.000	0.889	0.605	0.587
2. Forget things	0.301	1.000	1.000	1.000	0.813	0.644	0.562	0.507	0.469	0.405	0.311	0.304
3. Trouble keeping up with schoolwork	0.773	0.510	0.517	0.758	1.000	1.000	1.000	1.000	1.000	1.000	0.830	0.795
4. Miss school – not well	0.550	0.261	0.267	0.376	0.436	0.476	0.497	0.511	0.519	0.533	0.553	0.554
5. Miss school – doctor appointment	0.935	0.445	0.452	0.645	0.762	0.847	0.898	0.931	0.950	0.957	0.942	0.973

Total number of uniform DIF items	7	4	4	6	7	7	7	7	7	7	7	7

*Note*: DIF: differential item functioning; LASSO: least absolute shrinkage and selection operator; OLR: ordinal logistic regression; *w*: weighting parameter; the bold numbers show the *p*-values for items that demonstrate a uniform DIF.

## Data Availability

The real data utilized to support the results of this study are available from the corresponding author upon request.
